# Nursing Faculty Shortage Impact on Nursing Students: A Descriptive Phenomenological Study

**DOI:** 10.1155/2024/1751942

**Published:** 2024-10-29

**Authors:** Asia Antig, Shaniah Arañez, Chariemae Cañazares, Daisy Palompon

**Affiliations:** College of Nursing, Cebu Normal University, Cebu City, Philippines

**Keywords:** nursing faculty shortage, quality of education, undergraduate nursing students

## Abstract

**Background:** The nursing education sector has felt the impact of the shortage of nursing clinical instructors (CI) or faculty members. This phenomenon became more profound with the pandemic experience along with the global shortage of nurses in the hospitals.

**Objective:** The study was conducted to explore the lived experiences on learning among undergraduate nursing students in a higher education institution amidst the nursing faculty shortage.

**Methods:** The study was undertaken using a descriptive phenomenological design with seven informants, using Colaizzi's approach for data analysis.

**Findings:** The findings of the study generated three main themes: disruptions in the learning process and platforms, responses to learning disruptions, and learners' call to action. It uncovered substantial disruptions in scheduled lectures, activities, and clinical rotations due to the nursing faculty shortage.

**Conclusion:** The findings underscore the critical need for immediate and comprehensive measures to address the nursing faculty shortage. Despite compensatory efforts by the institution, the impact on learning experience remains significant. This study calls for prompt and effective strategies to rectify the nursing faculty shortage, ensuring optimal learning experiences for student nurses.

## 1. Background

In the Philippine setting, the Bachelor of Science in Nursing (BSN) program is guided by program standards and guidelines which set forth the minimum requirements and standards for implementing outcome-based education (OBE). The regulatory standards specify ideal faculty-to-student ratio in the classroom and Related Learning Experience (RLE), the clinical training of students. Furthermore, the regulation includes limitations in the teaching load depending on the management roles of the clinical instructor (CI) or faculty in certain institutions as they are required to have consultation hours and other activities related to RLE instruction, research, and extension services [1]. The guidelines not only set the standards for the BSN program but also serve as evidence corroborating the existence of a nursing faculty shortage in higher education institutions (HEIs) in the Philippines. In this study, the terms faculty and CI are used interchangeably, referring to one who teaches and supervises nursing students in the classroom and clinical areas.

For the past years, the Philippines have been producing many nurses; however, for the last 5 years worsened by the pandemic, the country faces understaffing in local hospitals. Per report, the shortage of nurses in the Philippines is around 127,000 and is expected to grow by 2030 [2]. The shortage of practicing nurses in the Philippines not only affected the clinical areas but the academe as well [3–5]. As a persistent issue, the nursing faculty shortage in nursing schools has substantial implications for nursing knowledge and practice development [6]. Therefore, the intent to enunciate the encounters of student nurses on faculty shortages remains imperative and warrants exhaustive inquiry. Recognizing the profound impact of the nursing faculty shortage, it is vital to investigate the perspectives of the individuals involved with the said phenomenon. Shortage of faculty poses a barrier to health care delivery as this reduces the number of students to be admitted in nursing school thereby reducing the health human resources in nursing [7, 8]. Faculty shortage is a contributor to the country's nursing shortage as this affects the quality of nursing education by disrupting the nursing educational process [9, 10] and reduces access to nursing education [11]. Lack of faculty also contributes to the shortage of faculty caring which influence level of academic stress of students and their intent to graduate [12, 13]. While several pieces of literature describe the encounters of educators and academic leaders regarding the existing faculty shortage [3, 14, 15], less is known from the student nurses' perspective. Student nurses, as recipients and beneficiaries of nursing education, experience firsthand the effects of faculty shortage and serve as determinants of its direct consequences [16, 17]. This study aimed to explore the lived experiences of student nurses amidst the nursing faculty shortage.

Schools of nursing have posted vacancies in their faculty positions both as regular and part-time engagement. However, despite the intensive recruitment, there were a lot of vacancies due to lack of applicants. This reveals a substantial gap in the number of CI necessary to meet the required RLE supervision of nursing students. To adhere to the standard guidelines, the Colleges of Nursing need to recruit additional faculty members to facilitate the learning outcomes of the students.

Recognizing the profound impact of the nursing faculty shortage, it is vital to investigate the perspectives of the individuals involved with the said phenomenon.

Exploring the student nurses' encounters with the nursing faculty shortage using naturalistic inquiry, underpinned the exploration of the experiences of student nurses amidst the nursing faculty shortage through the process of gathering and analyzing narrative and subjective data [18].

## 2. Review of Related Literature

The current nursing faculty shortage poses a serious and ongoing threat to the global infrastructure of health professions' education [6]. The need for more qualified nursing instructors in the Philippines is strongly associated with several factors, including a nursing faculty advancing in age, less attractive faculty positions, and increased qualifications required for faculty appointments, driven primarily by a constrained pool of faculty members with doctoral-level qualifications, the reluctance of school administrations to hire full-time nursing instructors, and the availability of more financially rewarding job opportunities in hospitals as opposed to nursing schools [19, 20] The implications of the nursing faculty shortage extend beyond the realm of education and healthcare, leading to a ripple effect that worsens the shortage of nurses both in clinical settings and in the academe [11].

As the nursing faculty shortage continues to be a significant challenge in the healthcare industry, the perspectives of nursing program administrators and academic leaders regarding this issue have been explored [14, 15]. Jarosinski et al. [14] revealed that administrators from community colleges and universities in the mid-Atlantic struggle with the nursing faculty shortage as it contradicts their goals of teaching excellence, quality student outcomes, and faculty satisfaction. From a leadership perspective, Vandyk et al. [15] spoke of nursing faculty shortage concerning the demand, supply, and strategies employed. Existing faculty vacancies further exacerbate the workload of remaining educators and imperil the continuity of service.

In their respective studies, Vandyk et al. [15] and Jarosinski et al. [14] employed qualitative research approaches to investigate the shortage of nursing faculty from different perspectives. Vandyk et al. [15] utilized qualitative descriptive phenomenology, conducting semi-structured interviews to explore the viewpoints of academic leaders in Canada. Through careful analysis of transcripts, they identified initial codes, emerging categories, and final themes related to the nursing faculty shortage. Similarly, Jarosinski et al. [14] placed significant emphasis on the perspective of nurse program administrators and employed purposive sampling with a sample size of 24 determined through maximum data saturation. Interviews were conducted using a semistructured format, and data analysis employed a 7-stage Heideggerian hermeneutic approach, identifying four consistent emerging themes that were evident throughout all interviews and explored themes [14].

Boamah, Callen, and Cruz [21] highlighted the presence of evidence that underscores the persistent threat posed by nursing faculty to the capacity of educational institutions to effectively educate and produce a sufficient number of proficient registered nurses capable of delivering quality patient care. Nursing faculty shortage has shown to have far-reaching implications for the development of nursing practice and the preservation of nursing knowledge. According to AACN [11]; the shortage of faculty in nursing schools is placing limitations on student enrollment capacity, while the need for highly skilled registered nurses continues to escalate.

Despite widespread recognition of nursing faculty shortages in various countries, the issue remains relatively understudied and largely unaddressed within the context of the Philippines. Consequently, the nursing faculty shortage in the country has created a predicament that directly impacts the quality of nursing education. One notable consequence of this shortage is the decline of student enrollment in nursing schools as an effort to maintain the quality of nursing education, posing significant challenges in the delivery of high-quality education to aspiring nurses in the Philippines [4, 22].

In a similar vein, although numerous studies explore the viewpoints of faculty members and nurse program administrators concerning the shortage of nursing educators, there is a notable absence of research examining the perspectives of nursing students, who play a key role in the educational process. Therefore, further research examining the experiences and views of nursing students regarding the nursing faculty shortage in the Philippines is warranted. Such research could provide valuable insights that may inform evidence-based interventions to address the nursing faculty shortage and mitigate its impact on nursing education and the healthcare industry.

## 3. Methods

A descriptive phenomenological design was employed to examine human experiences through the accounts of the participants and to make meaning of the encounters of student nurses amidst the nursing faculty shortage [23, 24]. The study explored the experiences of the nursing students with faculty shortage on their learning processes, the effects of the nursing faculty shortage to the students and the reactions of the students that prompt actions. Significantly, the researchers underwent bracketing (epoché) to facilitate phenomenological reduction and ensure the liberation of researchers from preconceived notions. In the process of bracketing, the researchers made their personal journals acknowledging their biases in the process. Reflexive discussions among researchers and a research mentor also enhance the phenomenological reduction to minimize the researchers' biases. The researchers employed a purposive sampling method to select participants from each eligible year level to explore the lived experiences of nursing students amidst the nursing faculty shortage. Purposive sampling is an intentional selection of informants based on their ability to elucidate a specific theme, concept, or phenomenon [25]. This was utilized since the intention of the study was to understand the experiences of the nursing students in situations when nursing faculty is scarce. Seven participants were included in the study after maximum data saturation was achieved. The study's inclusion criteria were legal age, currently enrolled in BSN with exposure to both lectures and skills laboratories within classroom settings and clinical experience in RLE.

Data were gathered through key informant interviews using the semistructured interview guide. The interview guide is composed of the first part which contains the demographic information of the informants. The second part comprised a series of open-ended questions to ensure an extensive exploration of the participants' lived experiences amidst the nursing faculty shortage. The questions were organized in a logical order that facilitated easy follow-up and responses from the participants. Questions included were: *Kindly describe your experiences as a student nurse amidst the nursing faculty shortage*; *Describe in greater details the impact of the nursing faculty shortage on your experiences as a student nurse*; *What are specific instances or situations where you have noticed the impact of the nursing faculty shortage on your experiences as a student nurse*?; and *How did these specific instances or situations affect you as a student nurse?* A preliminary interview simulation was conducted to pilot test the interview guide and to assess the quality of the researchers' communication skills necessary for extracting significant data from the participants.

The data gathering was conducted by the researchers. Two of the trained researchers conducted the interview. The interviewers underwent trainings and interview simulations prior to the data gathering. Bracketing was also undergone by the researchers to facilitate minimizing biases. Prior to the interview, a letter of invitation was sent to the prospective participants indicating the questions to be asked and requesting for their consent to join the study. The informed consent was signed by the informants prior to the interview.

Written consent allowing the audio recording of the interview was obtained from the interview participants. Each of the interviews lasted on the average of 51 min and 21 s, with each of the interview duration varying depending on their responses. Subsequent interviews were conducted for the seven participants with a minimum of two interviews and maximum of four interviews. Informed consent was obtained every time an interview was conducted. Transcription of the interview followed for detailed analysis. Following the completion of the interview, participants received monetary incentives as gratitude for their time and effort.

Data analysis was done using the seven-step approach of Colaizzi's method [26]. Initially, the data gathered through semistructured interviews was manually transcribed verbatim into written text with each transcription being preceded by bracketing through transpersonal reflexivity. First, familiarization was done by repeatedly reading the interview transcripts to gain a profound understanding of the data. Three researchers repeatedly read the interview transcripts to gain a profound understanding of the data. Three researchers repeatedly read the interview transcripts to gain a profound understanding of the data. Second, the researchers extracted significant statements pertinent to the phenomenon from the accounts of the participants. To facilitate data organization, the researchers maintained a record of the statements in a separate sheet with their line and page numbers. Third, the researchers formulated meanings that emerged from the significant statements. Fourth, the researchers grouped the formulated meanings into theme clusters and aggregated interconnected theme clusters to evolve into emerging themes. Fifth, the researchers focused on developing an exhaustive description of the phenomenon through careful integration of findings, followed by a review of the transcript, theme clusters, and themes to ensure a thorough interpretation. Next, the researchers condensed the exhaustive description of the phenomenon to reveal and describe its fundamental structure. The researchers then returned the produced fundamental structure of the phenomenon to the participants for validation if such statements accurately depict their experiences of the phenomenon under investigation [27, 28].

The researchers ensured credibility, transferability, dependability, and confirmability through a multifaceted approach. Member checking conducted at the outset of subsequent interviews, ensured accuracy in content interpretations. The study's methodology and participant demographics were transparently presented to enhance transferability. Rich, detailed descriptions of the participants' experiences were presented by including direct quotes and narratives by the participants. Additionally, the researchers reconfirmed the accuracy of the findings with their own experiences with the participants to prevent any distortions in the findings. A meticulous audit trail, encompassing detailed process logs and regular consultations with the research adviser, ensured systematic documentation. Finally, bracketing was done during the data collection and analysis to increase neutrality. Neutrality was maintained subsequently through transpersonal reflexivity.

The study was issued an approval for its conduct from the Review and Ethics Committee of the HEI under study. Participants were recruited by the researchers through social media invitations sent out to nursing students. Students who responded to the invitations were contacted to obtain their full consent. Obtaining full consent from the participants was prioritized, ensuring their participation was voluntary and without pressure and coercion. Furthermore, the participants' anonymity was ensured through omission of personal and sensitive data. The data provided were kept confidential using a serial number system, data encryption on participants' data files, and two-factor authentication on researchers' email accounts.

## 4. Results

Seven participants were selected and interviewed based on the set of inclusion criteria. After careful analysis of the interview transcriptions, 153 significant statements were extracted, and three main themes emerged. These emergent themes were disruptions in the learning processes and platforms, responses to learning disruptions and learners' call to action (Table 1).

### 4.1. Theme 1: Disruptions in the Learning Processes and Platforms

Learning as a process is expected to flow as a continuous experience for the learners. As one of the factors that led to productivity and learning efficiency of students, this must be well planned and implemented. One can attain learning efficiency as part of a critical aspect of nursing education, when it is gauged on how much a student's performance improves and how quickly, considering the time, effort, or resources that are expended [29]. Students have distinct preferences on how they might best learn efficiently, and this can be attributed to factors like learning platforms employed [30], delivery of instruction used [31] and schedule of their classes [32]. Any abrupt changes in these factors might affect the learning of the student, particularly when they occur without adequate preparation and support [33].

The findings of the study showed that the nursing faculty shortage has a tangible effect on the learning efficiency of student nurses through the changes in learning platform, delivery, and schedule.

#### 4.1.1. Sudden Transitions

The abrupt shift from in-person clinical duties to alternative learning experiences due to clinical instructor unavailability has disrupted the typical learning environment for nursing students. Through these transitions, three participants felt a sense of uncertainty in their educational experiences, often experiencing recurrent challenges and instances of confusion due to the shift to alternative learning modes:

*“There are times when…, a Clinical Instructor (CI) is scheduled, but their schedule is meant for our duty, yet they are actually unavailable. As a result, we become confused, and we find ourselves suddenly shifted back to an online setting. We return to online class because it is still considered acceptable, so it remains as such.”* (Participant 091523, T4, SS72, P19-20, L418-422)

#### 4.1.2. Overwhelming Activities

Student nurses in the Philippines are often met with substantial workload to achieve the learning objectives of the nursing curriculum, struggling with a rigorous nursing program that sets them apart from other college programs [34]. While its possible connection to the nursing faculty shortage remains unclear to most students, the participants grappled with the increased workload independent of CI supervision:

*“During the first year, it was difficult to tell if there was an unavailability of CIs because there are so many activities, so you don't know if they really had those activities to make up for the lack of CIs or their availability or if that was really what was required.”* (Participant 081823, T1, SS12, P12, L267-271)

#### 4.1.3. Frequent Learning Adjustments

Five out of seven participants experienced making frequent learning adjustments due to different clinical instructor during RLE, emphasizing the inconsistency and need for constant adaptation:

*“There was one week where we had three different clinical instructors for each day in three days and we had to adjust to all three of them … they're not really consistent because of the different styles of the instructors per week so it's almost as if every time we get a new Clinical Instructor, it's another adjustment that we have to cater to.”* (Participant 081823, T1, SS3, P7-8, L168-170,177–181)

Participants encountered challenges in scheduling changes and cancellations:

*“The same problems happen, but to a different degree, like, for example Monday, we start with this CI, but then, the next day, something came up, so we have to switch to a different CI… So, everything we have learned with the steps and procedures according to the standards of our first CI would have to be switched with the ones of the current CI, like, we have to learn another set of standards, so sometimes, we encounter such problems.”* (Participant 1012231, T6, SS127, P14, L284-291)

Some participants also found it difficult to adapt to different CIs' teaching approaches, as each CI had their own way of handling simulations and evaluating performance:

*“And then, for me, it's kind of difficult, because what I've realized is that each CI, they have their own way of handling the simulation process, different ways [of the simulation] to teach… In finding another CI to supervise, we had to adjust again because their way is different, so they would see our performance differently, and then the process would be different, and what we have learned so far would need to change so it could tailor fit the CI's standard.”* (Participant 090823, T3, SS55, P10, L221-233)

#### 4.1.4. Disruption and Cancellations of Learning Activities

All participants highlighted disruptions and cancellations of scheduled learning activities due to unavailability and overlapping schedules of CI. Instances that they could only attend one class out of multiple scheduled classes cause a lack of continuity in their learning experience:

*“There are days when we would come to school and three classes were scheduled for that day, but we could only attend one class because the clinical instructor for that subject would either be busy or unavailable. As a result, our day would be cut short.”* (Participant 081823, T1, SS5, P8, L192-196)

The lack of comprehensive discussions and uncovered topics, along with limited time available for learning pose significant challenges for effective knowledge absorption and skill development in the clinical setting:

*“It has also happened before that because of the unavailability or overlapping schedules of the clinical instructors, we were really catching up with the topic and there were a lot of topics and lessons that remained uncovered or were for just independent learning or asynchronous learning and some just get rushed through … It's a bit challenging for us to absorb the information because of the lack of time and the lack of comprehensive discussions.”* (Participant 081823, T1, SS9, P10, L230-235, 243–245)

Despite these challenges, however, participants acknowledged the quality of their CI and their meaningful discussions; hence, they expressed disappointment that the limited availability or shortage of CI often hindered learning opportunities, especially in terms of feedback, suggestions, comments, and tips:

*“I can really say that our clinical instructors are really good, and I've had many experiences where it's like really good discussions and everything. It's a shame that a lot of that time gets affected because of the lack of availability or shortage.”* (Participant 081823, T1, SS17, P13, L314-317)

### 4.2. Theme 2: Learners' Responses to Learning Disruptions

Learning flow is a state wherein students are fully immersed and engaged in their learning activities [35]. Changes to a new learning platform, delivery method, or schedule may introduce a significant interruption to the accustomed mode of instruction, causing a break in the continuity of the student's learning flow [35]. While some students adeptly navigated the ever-changing educational format, it proved to be a more challenging environment for those who thrive on structure and familiarity [36], resulting in negative responses.

The changes in learning platform, delivery, and schedule resulting in the negative responses of students were developed through the subthemes: (a) Dissatisfaction and Frustration and (b) Perceived Ineffective Process.

#### 4.2.1. Confusion, Overwhelmed, Uncertain, and Hindered Learning

Disruptions of learning activities, which were attributed to the nursing faculty shortage, adversely affected the participants' motivation and engagement. All participants in the study expressed their confusion and dissatisfaction resulting from these disruptions:

*“There was also no prior notice that we will not have class…we were just told that we won't have a … It feels such a loss because we went to school prepared … it's challenging and tiring”* (Participant 081823, T1, SS8, P9, L201-209)

*“For me, it's genuinely disappointing, and it really is pitiful on the part of the students.”* (Participant 101223, T5, SS118, P26, L570-572)

Some participants also expressed that they felt behind with other RLE groups, especially to those who did not have any disruptions in their learning activities, leading to feelings of envy and desire for the same learning opportunities as other RLE groups:

*“So, that's it, sometimes, there are moments when I feel envious. Personally, because, in the other groups, they have their own respective clinical instructor who is not that busy, whereas in our group, we were always left behind.”* (Participant 090823, T3, SS51, P8, L184-187)

With the cancellations or disruptions of learning activities due to unavailability of CI, some student nurses experienced being supervised together with other RLE groups under one clinical instructor. They expressed feelings of envy and frustration, especially with the quality and pace of return demonstrations under a clinical instructor supervising a significant number of students.

#### 4.2.2. Perceived Ineffective Process

Alternative measures were often implemented to mitigate disruptions of learning activities. All participants reported experiences of alternative measures, including the use of modules and independent learning, in the absence of their CI. These alternative measures implemented to mitigate disruptions of learning activities were perceived as not effective by all student nurses. They felt that these did not adequately compensate for the missed discussions and resulted in a less comprehensive learning experience:

*“What happens as a result is that we can only learn from ourselves, but I don't think it's sufficient in the long run. It would be much better if there were comments from the CIs.”* (Participant 083123, T2, SS34, P15, L314-316)

Furthermore, the participants expressed dissatisfaction and loss of motivation with these alternative measures, stating that they did not provide the same level of guidance and support as direct instruction from CI:

*(When asked how the substitution of CI affected his learning) “Personally, it greatly affected it [learning] because I lost the motivation to listen. Hence, because I'm not in the mood to listen, I wasn't able to absorb the lessons, so in the end, come midterms and finals, I had to catch up with the lessons in which I needed to do my own research …”* (Participant 090823, T3, SS45, P6, L120-121, 124–129)

Another alternative to mitigate the absence of assigned CI and continue learning activities was through substitute CI who were available. Modules were handed over to another CI to conduct the lesson. While some student nurses expressed that the substitute clinical instructor struggled to explain the lesson effectively, they acknowledged that this difficulty was not the fault of the substitute CI as the topic might be outside their expertise.

*“They … just give out their modules and hand them over to another CI so they can teach it, which was also kind of unfortunate. It's not the other CI's fault, but I gotta say that he won't be able to explain the lesson well and end up just reading the module since he's not also that familiar with the lesson. To be fair, though, it's another CI's topic.”* (Participant 090823, T3, SS44, P6, L106-109)

### 4.3. Theme 3: Learners' Call to Action

Clinical learning is essential for developing professional skills and preparing nursing students for their role as registered nurses [37]. Under the guidance of CI, lectures, skills laboratories, and clinical duties are perceived as more comprehensive and effective by students. Their call for action to address nursing faculty shortage was developed through the subthemes: Call to Stop Undesirable Effects, and Call to Address Nursing Faculty Shortage.

#### 4.3.1. Call to Stop Undesirable Effects

Two out of seven participants discussed their concerns about the implications of the nursing faculty shortage on the nursing graduate outcomes. One student nurse expressed apprehension about how the nursing faculty shortage might impact the quality of their education, and consequently, their preparedness for professional practice and future success as nurses:

*“First of all, it affects my confidence, because even when I graduate, I still won't know what to do. Like for example, in this exposure. We were able to try having this specialization exposure in this hospital, but not able to in another hospital. It was really a missed opportunity. My confidence really gets shaken up and I feel I'm not knowledgeable enough by the time I graduate if I haven't been able to experience those exposures.”* (Participant 101223, T5, SS112, P19, L413-417, 420–422)

Another student nurse echoed these concerns by highlighting the long-term consequences of limited exposure and constrained learning during the simulation and practice stages of their education:

*“So, if our learning is affected as early as now, during the simulation and practice stages, and with limited exposure, it will eventually build up to be our foundation as nurses working in the professional field.”* (Participant 1012231, T6, SS147, P31, L662-665)

#### 4.3.2. Call to Address the Nursing Faculty Shortage

Four out of seven student nurses called for action to address the ongoing nursing faculty shortage:

*“Quantity wise, I would say that maybe it's a seven out of ten rating like there really is room for improvement in terms of how many CIs there are.”* (Participant 1012231, T6, SS142, P28, L607-608)

The participants emphasized the need for consistency in the scheduling and availability of CI as it would result in a more comprehensive learning experience and enriching educational environment to both the students and CI. This stressed the importance of addressing the issue for the benefit of future batches, hoping to prevent them from encountering the same difficulties:

*“I'm hoping there would be more consistency in terms of the scheduling and availability of the CIs because it has been hectic.”* (Participant 081823, T1, SS15, P13, L307-309)

*“… This way, future batches won't have to go through the same challenges, and they can have a generally better experience, making them better nurses than us, which is our ultimate goal. Yes”* (Participant 1012231, T6, SS148, P31, L665-669)

In the absence of CI, the participants desired for well-thought backup plans and contingency measures to the challenges posed by faculty shortage:

*“There should be backup plans or contingency plans in place by the faculty, right? So, to compensate, and if they do compensate, maybe it should be done in a way that it's not substandard. Or, if they do compensate, it shouldn't be something that adds a lot of stress in the compensation process.”* (Participant 1012232, T7, SS162, P33, L671-675)

### 4.4. Exhaustive Description

The lived experiences of nursing students revealed how the nursing faculty shortage has significantly impacted the learning efficiency of nursing students by alterations across learning platforms, delivery methods, and schedules. These disruptions have deeply affected the learning experiences of nursing students, triggering feelings of disappointment, and diminishing motivation among them. The students stress the pivotal importance of nurturing fundamental nursing skills, maximizing learning opportunities, and completing required cases. To the students, these measures fail to adequately compensate for missed learning opportunities or offer a comprehensive educational experience. The nursing faculty shortage has evolved into a pervasive concern, garnering significant attention from students who are increasingly cognizant of its substantial impact on the quality of their education. The foresight among student nurses is particularly pronounced as they anticipate the long-term repercussions on the nursing profession, emphasizing how the shortage compromises the caliber of education, leading to the graduation of inadequately prepared and less confident nursing professionals. Students, accentuating the urgency conveyed, are not only calling for remedies but also expressing profound emotions, signifying the substantial weight the nursing faculty shortage carries within the educational landscape.

## 5. Discussion

The findings of the study showed how the shortage of nursing faculty disrupted the learning processes of the students. This has led to the variety of negative experiences from the learners, that led to their suggested resolutions to the shortage. The shortage of nursing faculty has generally precipitated several challenges, significantly influencing the education of student nurses. CI facilitate student nurses in achieving learning objectives and fostering active engagement [38]; thus, in their absence, student nurses may face difficulties in navigating the complexities throughout the nursing program, potentially leading to gaps in their knowledge and skill development [39]. As the primary stakeholders in their nursing education, student nurses directly feel the complex effects of this faculty shortage and therefore, can provide a lived account of its challenges. As students primarily experience the effects of the nursing faculty shortage, they are able to determine the effectiveness of mitigation efforts of the schools, and the overall quality of their educational experience amidst the phenomenon.

The pivotal role of CI in fostering essential knowledge and fundamental skills in undergraduate nursing students becomes evident in the face of these challenges. Consequently, studies have emphasized the critical importance of effective clinical instruction in achieving positive clinical outcomes, encompassing expanded knowledge, enhanced critical-thinking skills, and heightened confidence among students. In the context of the ongoing nursing faculty shortage, such faculty involvement takes on particular significance on the academic journey and overall satisfaction of student nurses [40, 41].

The nursing faculty shortage has led to limited exposure to clinical learning and simulations, which previous studies have linked to reduced skill development or unsatisfactory skill acquisition [42, 43], raising fears among students about their competency and knowledge adequacy as future nurses [44]. Moreover, clinical learning contributes to the students' professional development and motivation to graduate and continue in the nursing profession [13, 45]. To achieve effective clinical learning outcomes, continuous supervision from the CI during the clinical exposures of nursing students is crucial [46]. These findings highlight the need for proactive measures to address the challenges posed by the nursing faculty shortage to ensure the competency and confidence of future nursing graduates entering the professional field.

Addressing the effects of faculty shortage can be initiated through the advocacy, educational partnerships, and academic innovation [47]. There is a need for academic heads to advocate for quality education to allow innovative strategies on reinforcing students' learning are needed. The use of technology to expand the reach of nursing education through virtual and online classes can fill in the gap for lack of faculty [48]. The use of an online and blended teaching environment as well as collaborating with another college or university is among the strategies that can help minimize the negative effects of faculty shortage [12]. Educational partnerships allow resource sharing of faculty from other schools of nursing or hospitals. Preceptorship of qualified staff nurses in hospitals can serve as mentors in the clinical exposure of students [49].

The learners' call for action, revealed how student nurses have concerns about the potential long-term consequences of the shortage of nursing faculty on the professional practice and development of newly graduated nurses. This urgently necessitates collaborative efforts from administrators and CI to address the ongoing shortage of the nursing faculty. For the cultivation of a robust foundation in future professional nurses, consistent exposure to simulations and participation in clinical learning play a crucial role in honing decision-making abilities and fostering the development of professional skills [50].

The call for action to address the nursing faculty shortage emphasizes the far-reaching impact of the nursing faculty shortage on nursing education. It resonates with existing research on how the shortage's effect on nursing education has consequently impacted the healthcare system. Beyond the educational sphere, the shortage of qualified nursing faculty has broader implications, limiting the number of nursing students admitted and adequately educated [11]. This effect can exacerbate the existing shortage of healthcare professionals in the workforce due to the enduring impact of faculty shortage on educational institutions' ability to produce enough proficient registered nurses capable of delivering high-quality patient care [21].

The effects on the learning of the students brought about by the faculty shortage need to be a focus of research to determine its impact. Moreover, the use of new learning innovations needs to be studied on how this contributes to the learning of the students. Comparative studies on the competencies of nursing students affected by faculty shortage are an opportunity that can help in shaping policies of nursing academe.

### 5.1. Limitations of the Study

This study was conducted within a specific higher education institution in a city with predefined criteria and parameters. Thus, the experiences of student nurses in different institutions may differ in the context of the nursing faculty shortage. Additionally, despite the researchers' efforts to bracket and reflect on their preconceptions and biases regarding the nursing faculty shortage, there remains an acknowledgment of the possibility of these biases subtly influencing the study's findings. Despite attempts to neutralize these biases, their potential impact cannot be entirely ruled out.

## 6. Conclusion and Recommendations

Shortage of faculty in nursing has significantly affected the learning and confidence of students leaving them with disappointments in their experiences. This overlap has resulted in significant disruptions to the learning efficiency and overall educational experience of the students. Consequently, the disruptive nature of the nursing faculty shortage has led to feelings of discontent and frustration among the student nurses. Despite the educational institution's efforts to address the lack of CI through alternative measures, these interventions are perceived by students as inadequate substitutes for the traditional hands-on experience with CI and their expertise. In response to the ongoing nursing faculty shortage, student nurses are not only expressing dissatisfaction but also articulating a vital call to action, urging proactive measures to safeguard the competency and confidence of future nursing graduates, and thereby contributing valuable insights to enhance the overall quality of nursing education.

Encouraging supportive interventions to address nursing faculty shortage challenges is recommended. These interventions include allocating additional resources for online learning platforms, ensuring quality assurance for alternative teaching methods, and providing comprehensive guidance to students during critical periods. Moreover, implementing structured schedules for CI, improving recruitment and retention strategies for faculty, and fostering a supportive academic environment are suggested. Enhancing communication among faculty members and collaborating with government bodies to review and align the nursing curriculum with best practices are essential. Future research can aim to broaden participant and institutional scope and continuously survey nursing students to understand the impact of faculty shortage on their education and postgraduation competency.

## Figures and Tables

**Table 1:** Main themes and subthemes.

Themes	Subthemes
Disruptions in the learning processes and platforms	Sudden transitions
	Overwhelming activities
	Frequent learning adjustments
	Disruption and cancellations of learning
	Activities

Learners' responses to learning disruptions	Dissatisfaction and frustration
	Perceived ineffective process

Learners' call to action	Call to stop undesirable effects
	Call to address the nursing faculty shortage

## Data Availability

Data will be made available by the authors upon request.
